# Protective Role for Itaconate During Inhaled Allergen Challenge

**DOI:** 10.1111/all.70107

**Published:** 2025-10-24

**Authors:** Gesa J. Albers, Patricia P. Ogger, Christina Michalaki, Helen Stölting, Simone A. Walker, Anna Caldwell, John M. Halket, Kathryn Duvall, Atia Batool, Lisha Joshi, Helen O'Brien, Cormac McCarthy, Timothy Hinks, Gail M. Gauvreau, Paul M. O'Byrne, Clare M. Lloyd, Adam J. Byrne

**Affiliations:** ^1^ National Heart and Lung Institute Imperial College London London UK; ^2^ Department of Nutritional Sciences, School of Life Course & Population Health Sciences King's College London London UK; ^3^ Conway Institute and School of Medicine University College Dublin Dublin Ireland; ^4^ Respiratory Medicine Unit, Nuffield Department of Medicine and National Institute for Health Research Oxford Biomedical Research Centre University of Oxford Oxford UK; ^5^ Clinical and Experimental Sciences University of Southampton Faculty of Medicine, Sir Henry Wellcome Laboratories, and the NIHR Southampton Respiratory Biomedical Research Unit, Southampton University Hospital Southampton UK; ^6^ Department of Medicine McMaster University Hamilton Ontario Canada

**Keywords:** airway inflammation, allergen exposure, asthma, itaconate, macrophage metabolism

## Abstract

**Background:**

Asthma is a chronic, heterogeneous disease characterised by airway remodelling, inflammation, and mucus production. Airway macrophages' functions are underpinned by changes in cellular metabolism. The TCA cycle‐derived metabolite itaconic acid (whose synthesis is mediated by aconitate decarboxylase) is a master regulator of macrophage function; however, its role during inhaled allergen challenge is not clear. The objective of this study was to define the role of itaconate during inhaled allergen challenge.

**Methods:**

Sputum metabolite levels were measured in participants with mild allergic asthma undergoing allergen inhalation challenge, and in a second cohort, baseline levels in mild, moderate, and severe asthmatics. Airway inflammation, lung function, and bronchoalveolar lavage metabolite levels were assessed in wild‐type and aconitate decarboxylase‐deficient mice, or in mice treated with inhaled itaconate.

**Results:**

Allergen inhalation in mild asthmatics led to a significant reduction in sputum itaconate. We found no difference in baseline sputum itaconate levels when comparing healthy controls to mild, moderate, or severe asthmatics. Continuous exposure to aeroallergen in wild type and aconitate decarboxylase‐deficient mice showed no change in disease phenotype after 48 h, 1, 3, or 5 weeks of allergen exposure. Treatment of house dust mite‐exposed mice with inhaled itaconate reduced airway inflammation.

**Conclusion:**

Levels of itaconate are altered after allergen challenge in mild asthmatics and in murine models of disease. Itaconate deficiency did not alter house dust mite‐induced pathology at any of the timepoints tested; however, inhaled itaconate ameliorated inflammatory responses to inhaled allergen.

## Graphical abstract

Itaconate levels decrease in sputum following allergen inhalation in mild asthmatics, suggesting a dynamic metabolic response to airway challenge. Genetic deletion of *Acod1* in mice did not alter airway inflammation or remodelling in response to chronic HDM exposure. Inhaled itaconate treatment reduced airway inflammation in HDM‐exposed mice, highlighting its potential as a therapeutic strategy for allergic airway disease. Abbreviations: *Acod1*, aconitate decarboxylase; AMs, airway macrophages; BAL, bronchoalveolar lavage; *Ccl5*, C‐C motif chemokine ligand 5; Ctrl, control; *Cxcl1*, C‐X‐C motif chemokine ligand 1; DCs, dendritic cells; HDM, house dust mite; Tgfb1, transforming growth factor beta 1; WT, wild type.

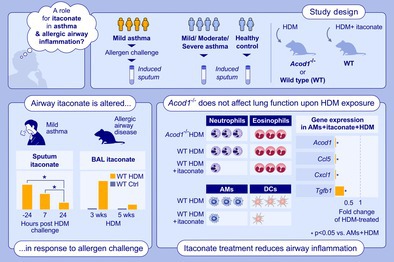

**Abbreviations**
AAD: allergic airways disease
AHR: airway hyperresponsiveness
AMs: airway macrophages
BAL: bronchoalveolar lavage
BMDMs: bone marrow‐derived macrophages
BMI: body mass index
cDNA: complementary DNA
DCs: dendritic cells
EAR: early asthmatic response
EDTA: ethylenediaminetetraacetic acid
ELISA: enzyme‐linked immunosorbent assay
FACS: fluorescence activated cell sorting
FEV₁: forced expiratory volume in one second
FMO: fluorescence minus one
FVC: forced vital capacity
GC–MS: gas chromatography–mass spectrometry
HDM: house dust mite
HRP: horseradish peroxidase
i.m.: intramuscular
i.n.: intranasal
i.p.: intraperitoneal
IA: itaconic acid
ICS: inhaled corticosteroids
IgE: immunoglobulin E
IgG1: immunoglobulin G1
IPF: idiopathic pulmonary fibrosis
LAR: late asthmatic response
MCh: methacholine
Na_2_EDTA: sodium ethylenediaminetetraacetic acid
NRF2: nuclear factor erythroid 2‐related factor 2
OVA: ovalbumin
PBS: phosphate‐buffered saline
PC_20_
: provocative concentration causing a 20% drop in FEV₁
PMA: phorbol 12‐myristate 13‐acetate
qPCR: quantitative polymerase chain reaction
RLT: RNA lysis tissue buffer
RNA: ribonucleic acid
SD: standard deviation
SDH: succinate dehydrogenase
(TCA) cycle: tricarboxylic acid
(Th2) cells: T helper 2
VC: vital capacity
WT: wild type

## 1 Introduction

Asthma is a heterogeneous disease of airways characterised by variable airflow obstruction, airway hyperresponsiveness (AHR), and inflammation [1]. Although most patients achieve good control with conventional asthma therapies (inhaled corticosteroids and bronchodilators), a proportion experience life‐threatening, severe disease [2]. Evidence suggests that T helper 2 (Th2) cells and their cytokines orchestrate allergic airway inflammation, which leads to airway remodelling and lung dysfunction [3]. However, despite systemic sensitization to airborne allergen, not all allergic individuals develop asthma upon allergen exposure [4, 5, 6]. Furthermore, some studies suggest that airway inflammation is similar in atopic and non‐atopic asthma, with overlapping infiltration of eosinophils, mast cells, and lymphocytes [7]; while other evidence highlights distinct inflammatory profiles [3], including increased eosinophils in the airways of atopic asthmatics and neutrophils in non‐atopic asthma [8, 9]. These findings suggest that while type 2 immunity is a common feature in both atopic and non‐atopic asthma, it may be necessary but not sufficient to define the allergic asthmatic phenotype, and additional immune or environmental factors may contribute to observed clinical differences.

The role of specific metabolites in regulating asthma phenotypes remains poorly understood. While numerous studies have examined changes in metabolite levels in serum samples between individuals with and without asthma, data from primary airway samples has been relatively limited [10, 11, 12]. Activation of macrophages with a range of pro‐inflammatory stimuli induces a metabolic switch from oxidative phosphorylation to glycolysis, similar to the ‘Warburg effect’ seen in some cancers [13]. Conversely, activation of macrophages in vitro with type 2 cytokines drives a highly oxidative metabolic phenotype, characterised by high mitochondrial metabolism and elevated oxidation of the tricarboxylic cycle (TCA) intermediates [14, 15]. Itaconate (IA), a TCA cycle metabolite synthesised by cis‐aconitate decarboxylase (CAD) encoded for by the gene *ACOD1* [16], has emerged as a central factor in the regulation of macrophage function and phenotype. In bone marrow‐derived macrophages (BMDMs), itaconate is one of the most highly upregulated metabolites following stimulation with LPS [17]. While itaconate has been shown to fine‐tune macrophage metabolism by inhibition of the TCA cycle enzyme succinate dehydrogenase (SDH), it is also immunomodulatory [18]. For instance, itaconate can function as an anti‐inflammatory molecule via alkylation of Kelch‐like ECH‐associated protein 1 (KEAP1), driving the accumulation of nuclear factor erythroid 2‐related factor 2 (NRF2), which can translocate to the nucleus and initiate the transcription of anti‐inflammatory genes [17]. Recently, we reported that in murine models of bleomycin‐induced pulmonary fibrosis, and patient samples from patients with idiopathic pulmonary fibrosis (IPF), itaconate is antifibrotic [19]. Recent studies have shown that in mice, itaconate or *Acod1* expression may be protective [20, 21, 22, 23]; however, the role of itaconate across specific asthma phenotypes, as well as during human allergen exposure, is yet to be determined.

In this study, we aimed to examine the role of itaconate in human asthma and AAD under conditions of low‐dose, continuous exposure to a relevant aeroallergen (house dust mite, HDM). Our findings reveal that allergen exposure in sensitised individuals with mild asthma leads to a rapid decrease in sputum itaconate levels. However, we observed no significant differences in sputum itaconate concentrations among mild, moderate, or severe asthmatics compared to healthy controls. In our AAD model, *Acod1* deficiency did not affect disease severity, with cellular infiltration, lung function, and other disease markers remaining unaltered by the loss of *Acod1*. While inhaled exogenous itaconate induced modest shifts in inflammatory infiltrates in AAD, with an amelioration in pulmonary infiltration, including neutrophilia. Collectively, our data suggest a role for itaconate during human allergen exposure; however, murine data indicate that this role may be highly context‐dependent.

## 2 Methods

### 2.1 Human Sputum Samples

#### 2.1.1 Allergen Challenge Cohort

Cell pellets and supernatants of human sputum samples of 10 patients with mild asthma were received from McMaster University, Canada. Participating male and female patients between 18 and 55 years were screened according to patient inclusion and exclusion criteria as shown in Table S1. For allergens with seasonal variation, patients were tested out of season for pollens affecting their asthma; all patients had no other lung diseases. No asthma‐controller treatments were allowed during the study; however, the use of inhaled short‐acting β2‐agonists, administered fewer than 2 days per week as rescue treatment, was permitted. All other asthma medications were discontinued at least 4 weeks before enrollment. Patients were excluded from the study if they had worsening of asthma or respiratory‐related visits to the emergency department within 6 weeks before study enrollment. To choose the allergen for an inhaled allergen challenge, the patients' sensitising allergen was determined using a skin prick test. The methacholine inhalation test was performed to ensure that only patients with asthma‐like responses were included in the study cohort and was carried out by the standardised 2‐min tidal breathing technique [24]. Participants underwent a minimum washout period of 48 h between the methacholine challenge and the allergen inhalation challenge. Allergen was administered from a Wright nebuliser and inhaled for 2 min. The optimal concentration of allergen was determined as the lowest dilution of allergen inducing a skin wheal of 2 × 2 mm and producing a 20% early asthmatic response (PC_20_) calculated using a formula described in Cockcroft et al. [25, 26]. We performed allergen and methacholine challenges and collected venous blood and sputum samples via standard methods [27] for subsequent analysis. Analysis of samples from this cohort has been previously reported; a secondary analysis of these samples was conducted in the present study [28, 29].

### 2.2 Mild/Moderate/Severe Asthma Cohort

Induced sputum was collected from 37 participants (20–67 years), enrolled from the National Institute for Health Research Southampton Respiratory Biomedical Research Unit and outpatient clinics at University Hospital Southampton (REC number 10/H0504/2) as previously described [30]: 10 healthy non‐atopic participants, six mild patients with asthma on β2‐agonists alone, eight subjects with moderate asthma on ICS, and 13 subjects with severe asthma with persistent symptoms despite high‐dose ICS and oral corticosteroids (cohort characteristics have recently been reported [30]), classified on enrolment by applying criteria used previously. The study was approved by the Southampton and Southwest Hampshire Research Ethics Committee B. All participants provided informed consent.

### 2.3 Sputum Processing

Sputum cells were pelleted by centrifugation, resuspended in 0.2% dithiothreitol, and filtered through 48 μm nylon mesh. Sputum supernatants were aliquoted and stored at −80°C for targeted gas chromatography–mass spectrometry analysis.

### 2.4 Experimental Animals

Wild type (WT) female C57BL/6 mice were purchased from Charles River Laboratories or WT male or female littermates obtained from own breeding colonies bred at Charles River Laboratories and housed at Imperial College Central Biomedical Services facilities for experimental procedures. Mice included in these studies were utilised specifically to delineate the role of itaconate during AAD. Female *Acod1* ^*−/−*^ mice and controls were on a C57BL/6 background and were purchased from Jackson Labs. All mice used in in vivo experiments were female. Both male and female WT mice were used for ex vivo treatment of airway macrophages. The UK Home Office guidelines under the Animal (Scientific Procedure) Act 1986 were followed during all experiments, and experiments were approved by the Imperial College London Welfare and Ethical Review Body (AWERB) and the Home Office of the United Kingdom. Mice were sacrificed by intraperitoneal (i.p.) injection of pentobarbital and severing a peripheral artery unless used for assessment of lung function. All surgery was performed under anaesthesia using ketamine and sodium pentobarbital, and all efforts were made to minimise suffering.

### 2.5 Gene Expression Analysis

AMs obtained from the BAL of naïve male and female C57BL/6 mice were exposed to 10 mM itaconate (Sigma Aldrich) for 3 h followed by 50 μg/mL house dust mite (HDM; *Dermatophagoides pteronyssinus*; batch 15J02, Citeq Biologics) for 12 h. HDM‐exposed AMs or the post‐caval murine lung lobe were lysed in RLT buffer (Qiagen) supplemented with 1% 2‐mercaptoethanol and the murine post‐caval lung lobes were homogenised with Lysing Matrix D beads and a FastPrep‐24 homogeniser (MP Biomedicalws). Total RNA was isolated using the RNeasy Plus Micro (AMs) or Mini (lung tissue) kit (Qiagen) according to manufacturer's instructions. For gene expression analysis by quantitative real time polymerase chain reaction (qPCR), cDNA was generated from total RNA using the High‐Capacity cDNA Reverse Transcription kit (Thermo Fisher) as per manufacturer's instructions and a PTC‐200 Peltier Thermal Cycler (MJ Research). qPCR was performing using TaqMan Fast Advanced Master mix and primer probes (Thermo Fisher; *Actb*: Mm00607939_s1, *Acod1*: Mm01224532_m1) as per manufacturer's instructions and assays were run in 384‐well plates on a Viia7 instrument. Gene expression relative to housekeeping gene *Actb* was calculated as $2^{-\mathrm{dCT}}.$

### 2.6 Metabolic Flux Analysis

AMs obtained from the BAL of naïve female *Acod1* ^*−/−*^ mice and WT littermate controls were exposed to 50 μg/mL HDM (Dermatophagoides pteronyssinus; batch 15J02, Citeq Biologics) for 24 h. Oxygen consumption rate (OCR) and extracellular acidification rate (ECAR) were measured using the Cell Mito Stress Test kit and the Glycolysis Stress Test kit (both Agilent Technologies) as per manufacturer's instructions and as previously described [19, 30, 31], OCR and ECAR measurements were made with an XFp Extracellular Flux Analyzer (Agilent Technologies) and results were analysed with Wave software version 2.6.0 (Agilent Technologies).

### 2.7 In Vivo Mouse Models

To induce AAD in WT mice, mice were instilled with 25 μg HDM (*Dermatophagoides pteronyssinus*; Citeq Biologics, batch 15J02) or 25 μL phosphate‐buffered saline (PBS; Gibco) intranasally (i.n.) while anaesthetized using isoflurane three times a week over the course of 1, 3, or 5 weeks. To evaluate the effects of exogenous itaconic acid (IA) administration during AAD, mice were administered 5 μg itaconate (Sigma Aldrich) in 25 μL PBS or 25 μL PBS i.n. in addition to HDM treatment. WT mice were treated with inhaled itaconate twice weekly either over a period of 3 weeks or during the last 2 weeks of a five‐week continuous exposure experiment.

### 2.8 Lung Function Measurements

Mice were anaesthetised by i.p. injection of 50 mg/kg pentobarbitone (Sigma Aldrich) and intramuscular (i.m.) injection of 200 mg/kg ketamine (Fordodge Animal Health Ltd), and lung function measurements were performed using the flexiVent system (Scireq). Mice were tracheotomised and connected to the flexiVent ventilator via a blunt‐ended 19‐gauge needle and ventilated using a tidal volume of 7 mL/kg body weight, 150 breaths/min, and positive end‐expiratory pressure of approximately 2 cm H_2_O. The lung volume history was standardised with three deep inflations, and dynamic resistance (R) in response to increasing concentrations of nebulised methacholine (MCh) was calculated by fitting measurements obtained using the snapchot‐150 perturbation, a single frequency sinusoidal waveform, to a single compartment model. Changes in airway resistance (R), elastance (E), and compliance (C) were expressed as cmH_2_O × s/mL, cmH_2_O/mL, and mL/cmH_2_O, respectively.

### 2.9 Isolation of Immune Cells From BAL and Lung Tissue

Mice were tracheotomised, cannulated, and the airways were lavaged three times with 0.4 mL PBS/0.5 mM ethylenediaminetetraacetic acid (EDTA; Thermo Fisher), and approximately 1 mL of BAL was collected. BAL cells were pelleted by centrifugation, supernatants were collected, and cells were resuspended in cRPMI. The left lung lobe was finely chopped and incubated with cRPMI supplemented with 0.15 mg/mL Collagenase D (Roche Diagnostics) and 25 μg/mL DNase I (Roche Diagnostics) at 37°C for 1 h. The recovered cells were passed through a 70 μm filter to obtain a single‐cell suspension; erythrocytes were lysed using a 55 mM NH_4_Cl/10 mM KMCO_3_/0.1 mM Na_2_EDTA lysis buffer, pelleted, and resuspended in cRPMI.

### 2.10 Flow Cytometry

BAL and lung cells were stained with near‐infrared fixable live/dead dye (1:1000, Thermo Fisher) before staining for extracellular antigens in fluorescence‐activated cell sorting (FACS) buffer (1% FBS/25 mM HEPES (Thermo Fisher)/1 mM EDTA in PBS). Cells were fixed using IC Fixation buffer (Thermo Fisher). For panels including intracellular cytokine staining, cells were stimulated with 20 ng/mL phorbol 12‐myristate 13‐acetate (PMA; Sigma Aldrich), 1.5 μg/mL ionomycin free acid from *Streptomyces conglobatus* (Merck), and 5 μg/mL Brefeldin A (Sigma Aldrich) for 4 h prior to extracellular antigen staining. Following stimulation, staining for extracellular antigens and fixation, cells were permeabilised using permeabilisation buffer (eBioscience) and stained with intracellular antigens in permeabilization buffer; see Table 1 for details of antibodies, clones, and dilutions utilised in murine flow cytometry experiments. For flow cytometry cell sorting, BAL cells were stained for extracellular antigens diluted in low‐protein FACS buffer (0.1% BSA (Sigma Aldrich)/25 mM HEPES/1 mM EDTA in PBS). Immediately before acquisition, 50 μL/mL of 1 μM ToPro3 viability dye (Thermo Fisher) was added, and AMs were sorted into 10% FBS/25 mM HEPES in PBS. Data were acquired using a BD LSR Fortessa cell analyser and analysed using FlowJo (all BD Biosciences). Individual cell populations were gated using fluorescence minus one (FMO) controls, and gating strategies are shown in Figure S3 as described previously [19, 30].

**TABLE 1:** Antibodies, clones and dilutions utilised in murine flow cytometry experiments.

Antigen	Clone	Flurochrome(s)	Manufacturer	Dilution	Method
CD103	2E7	FITC	Biolegend	1:100	Extracellular
CD11b	M1/70	PE/Cy7	Biolegend	1:200	Extracellular
CD11c	N418	PE/Dazzle 594	Biolegend	1:100	Extracellular
CD19	6D5	BV605	Biolegend	1:100	Extracellular
CD3ε	145‐2C11	BV510	Biolegend	1:100	Extracellular
CD4	RM4‐5	AF700	Biolegend	1:200	Extracellular
CD8a	53–6.7	BV605	Biolegend	1:100	Extracellular
CD45	30‐F11	BV711	Bioloegend	1:200	Extracellular
CD64	X54‐5/7.1	BV421	Biolegend	1:100	Extracellular
CD90.2 (Thy‐1.2)	53–2.1	BV605	Biolegend	1:200	Extracellular
Fc Block (CD16/CD32)	2.4G2	Unconjugated	Tonbo	1:100	Extracellular
IFN‐γ	XMG1.2	FITC	Biolegend	1:100	Intracellular
IL‐5	TRFK5	PE	Biolegend	1:100	Intracellular
IL‐10	JES5‐16E3	FITC	BD	1:100	Intracellular
IL‐13	eBio13A	PE/Cy7	Thermo Fisher	1:100	Intracellular
IL‐17A	TC11‐18H10.1	PercCP/Cy5.5	Biolegend	1:100	Intracellular
Ly6C	HK1.4	AF700	Biolegend	1:200	Extracellular
Ly6G	1A8	BV510, BV605	Biolegend	1:100	Extracellular
MHC II (IA‐IE)	M5/114.15.2	PerCP/Cy5.5	Biolegend	1:100	Extracellular
NKp46 (CD335)	29A1.4	BV605	Biolgened	1:100	Extracellular
Siglec F	E50‐2440	PE	Biolegend	1:100	Extracellular

### 2.11 ELISA

Up to 500 μL of blood was collected from mice following 1 or 3 weeks of exposure to HDM, and the serum was separated using serum separator tubes (BD). Total serum titres of IgE and IgG1 were measured by enzyme‐linked immunosorbent assay (ELISA) using respective capture antibodies (all BD) diluted in 0.1 M NaHCO_3_, biotinylated detection antibodies (all BD), streptavidin‐conjugated horseradish peroxidase (HRP; R&D Systems), K‐blue substrate (Neogen), and 0.18 M H_2_SO_4_ to stop the colorimetric reaction. Absorbance was measured using a SpectraMax i3x reader (Molecular Devices).

### 2.12 Measurement of Metabolites by Targeted GC–MS

Metabolite levels in freeze‐dried murine BAL collected from mice following 1, 3, or 5 weeks of exposure to HDM or in vivo and human sputum supernatants of healthy individuals and subjects with atopic asthma were measured by targeted gas chromatography–mass spectrometry (GC–MS) as previously described [30].

### 2.13 Histology

The superior lobe of HDM‐exposed mice was inflated with PBS, fixed in 10% neutral buffered formalin, and embedded in paraffin. 4 μM sections were stained with Haematoxylin and Eosin (H&E) as well as Periodic Acid Schiff (PAS) staining. To assess cellular inflammation and immune cell infiltration within the lungs, H&E‐stained lung sections were scored from 0 (no inflammation) to 5 (highest levels of inflammation) based on the following criteria: 0 = no inflammation, 1 = small pocket of infiltrate, 2 = small pocket of infiltrate (< three cells deep) in at least two airways, 3 = less than 50% of airways have large infiltrates (> three cells deep), 4 = 50%–75% of airways have large infiltrates, and 5 = > 75% of airways have large infiltrates. Goblet cell hyperplasia was assessed in PAS‐stained sections using the following scoring system: 0 = no mucus staining, 1 = 5%–25% of airway stained, 2 = 25%–50% of airway stained, 3 = 50%–75% of airway stained, 4 = > 75% of airway stained. The scores were normalised to the number of airways assessed.

### 2.14 Statistics

All data processing, analysis and plotting was conducted in RStudio. A two‐sided Mann–Whitney test was used for statistical analyses comparing two groups and a Wilcoxon signed‐rank test was used to compare paired samples. *p* values < 0.05 were considered statistically significant. For correlations, exact *p* values and rank correlation coefficients (rho) were calculated using the Spearman method. Data was visualised using the ggplot2 package.

## 3 Results

### 3.1 Airway Itaconate Is Altered in Response to Allergen Challenge

**FIGURE 1:**
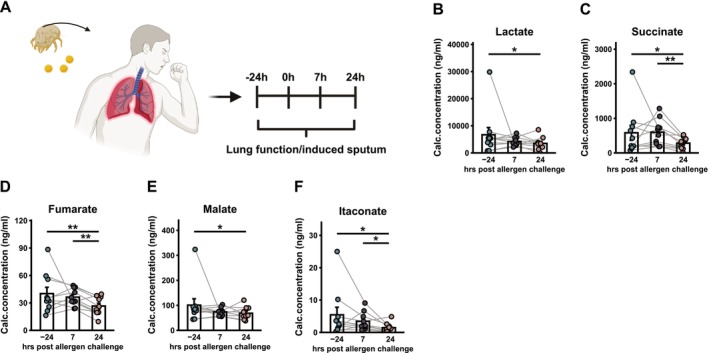
Altered sputum itaconate after allergen challenge. (A) Patients with mild asthma were challenged with sensitising allergen, and induced sputum was collected at indicated timepoints. Levels of (B) Lactate, (C) succinate, (D) fumarate, (E) malate, and (F) itaconate in sputum supernatants pre and post allergen challenge, as determined by targeted GC–MS. *n* = 10 matched samples. Data presented as mean ± SEM. *p < 0.05, **p < 0.01, Wilcoxon signed‐rank test.

To investigate metabolic changes in sputum of patients with mild asthma in response to allergen challenge, glycolysis and TCA cycle metabolite levels in induced sputum were measured by targeted GC–MS (Figure 1A, Figure S1A,B). Inclusion and exclusion criteria are detailed in Table S1 and Table 2 shows study subject demographics and allergen sensitivity. Ten non‐smoking subjects (two males, eight females; mean age 25.1 ± 6.8 years; BMI 24.2 ± 3.3 kg/m^2^) participated in the study. Baseline lung function was within normal limits (% predicted: 97% ± 12.4%), and mean early asthmatic response (EAR) was a 29.6% ± 11.2% fall in FEV_1_, while the late asthmatic response (LAR) showed a mean decline of 18.2% ± 10.2%. Analysis of sputum metabolite levels revealed significant changes in the airway metabolome following allergen challenge (Figure S1C, Figure 1B–F). Notably, there was a marked reduction in the absolute concentration of lactate, the end product of glycolysis (Figure 1B), along with key intermediates of the TCA cycle: succinate (Figure 1C), fumarate (Figure 1D), and malate (Figure 1E) at 7 or 24 h after allergen exposure, compared to pre‐challenge levels. When comparing pre‐ and post‐challenge, we found no difference in sputum levels of glucose (Figure S1D), glycerol‐1‐phosphate (Figure S1E), glycerol‐2‐phosphate (Figure S1F), pyruvate (Figure S1G), citrate (Figure S1H), cis‐aconitate (Figure S1I), or α‐ketoglutarate (Figure S1J). Interestingly, sputum itaconate was decreased at both 7 (2.01 pg/mL or 37.02% decrease) and 24 h (3.97 pg/mL or 73.11% decrease) post allergen challenge, compared to pre‐challenge levels (Figure 1F). In a second cohort, we assessed baseline levels of itaconate in sputum samples obtained from healthy controls, as well as mild, moderate, and severe asthmatics (Figure S1K). We found reduced citrate in mild asthmatics (Figure S1L), reduced cis‐aconitate in moderate and severe asthmatics (Figure S1M), and reduced isocitrate in severe asthmatics (Figure S1N), compared to controls. Interestingly, there was no difference in itaconate levels, regardless of asthma severity, compared to controls (Figure S1O). These findings suggest that metabolite levels in asthmatic sputum are strongly influenced by both disease severity and allergen exposure. Notably, alterations in itaconate levels are context‐dependent, with no differences observed between asthmatic and control airways at baseline. However, after allergen exposure, itaconate levels decrease significantly at both 7 and 24 h post challenge.

**TABLE 2:** Subject demographics (allergen challenge cohort).

	Mean ± SD
Subjects (*n*)	10
Age (years)	25.1 ± 6.8
Male:female (*n*)	2:8
BMI (kg/m^2^)	24.2 ± 3.3
Smoking:non‐smoking (*n*)	0:10
Lung function	
FEV_1_ (L/s)	3.6 ± 0.6
VC (L)	4.3 ± 0.8
FEV_1_/VC ratio	0.82 ± 0.18
FEV_1_% predicted	97 ± 12.4
EAR (% fall in FEV_1_)	−29.6 ± 11.2
LAR (% fall in FEV_1_)	−18.2 ± 10.2
Challenged with	
HDM (*Dermatophagoides farinae*) (*n*)	2
HDM (*Dermatophagoides pteronyssinus*) (*n*)	1
Ragweed (*n*)	1
Horse (*n*)	1
Timothy Grass (*n*)	1
Kentucky Blue Grass (*n*)	2
Cat (*n*)	2

*Note:* Table showing subject number, age, sex, body mass index (BMI), forced expiratory volume (FEV_1_), FEV_1_ predicted, vital capacity (VC), FEV_1_‐to‐VC ratio, early asthmatic response (maximum % fall in FEV_1_ 0–2 h post‐challenge), early asthmatic response (EAR, at least 20% decrease in FEV1 within 2 h of allergen inhalation), late asthmatic response (LAR, maximum % fall in FEV_1_ from 3 to 7 h post‐allergen) and the allergen extract that subjects were challenged with. Data presented as mean ± SD.

### 3.2 Altered Itaconate/Acod1in a Murine Model of Allergic Airways Disease

**FIGURE 2:**
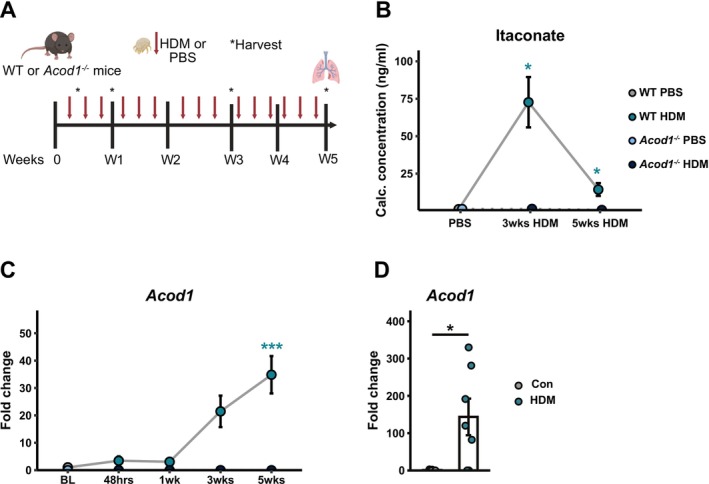
(A) Schematic of HDM exposure model. Metabolite levels in BAL of WT and *Acod1* ^*−/−*^ mice exposed to inhaled HDM for three or five weeks as measured by targeted GC–MS. (B) Itaconate levels in BAL of WT and *Acod1* ^*−/−*^ mice exposed to inhaled HDM for three or five weeks as measured by targeted GC–MS. (C) *Acod1* expression in whole lungs following HDM exposure relative to housekeeping gene *Actb* measured by qPCR. Significant differences between matched HDM‐ and PBS‐treated groups are indicated by stars in teal (WT PBS vs. WT HDM) or navy (*Acod1* ^*−/−*^ PBS vs. *Acod1* ^*−/−*^ HDM). Data shown from 1 to 2 independent experiments per timepoint with *n* = 3–8 mice per group per experiment. (D) *Acod1* expression in isolated WT airway macrophages following HDM exposure ex vivo, relative to housekeeping gene *Actb* measured by qPCR. Data shown from two independent experiments with *n* = 3–4 mice per group. Data presented as mean ± SEM. Mann–Whitney test, *p < 0.05, ***p < 0.001.

To determine the functional role of itaconate during AAD, we utilised a continuous, clinically relevant, allergen exposure model. *Acod1* expression has been shown to be upregulated in response to HDM in murine lung in DCs [23] and AMs [21]. Consistent with these reports, in our model, repeated exposure to inhaled HDM in WT mice (Figure 2A) led to significant alterations in the lung metabolome (Figure S2A,B), including elevated levels of BAL itaconate (Figure 2B), as measured by targeted GC–MS. Itaconate levels remained consistently elevated, compared to controls, throughout the five‐week course of HDM administration and peaked after 3 weeks of challenge (Figure 2B). This increase in BAL itaconate was accompanied by upregulated *Acod1* expression in whole lung tissue following HDM exposure (Figure 2C).

Similarly, the challenge of isolated airway macrophages (AMs) with HDM also resulted in heightened *Acod1* expression (Figure 2D). Thus, chronic HDM exposure induces sustained increases in itaconate levels in the lung and enhanced *Acod1* expression.

### 3.3 Acod1 Deficiency Does Not Alter Lung Function or Innate Immunity During Continuous HDM Exposure

**FIGURE 3:**
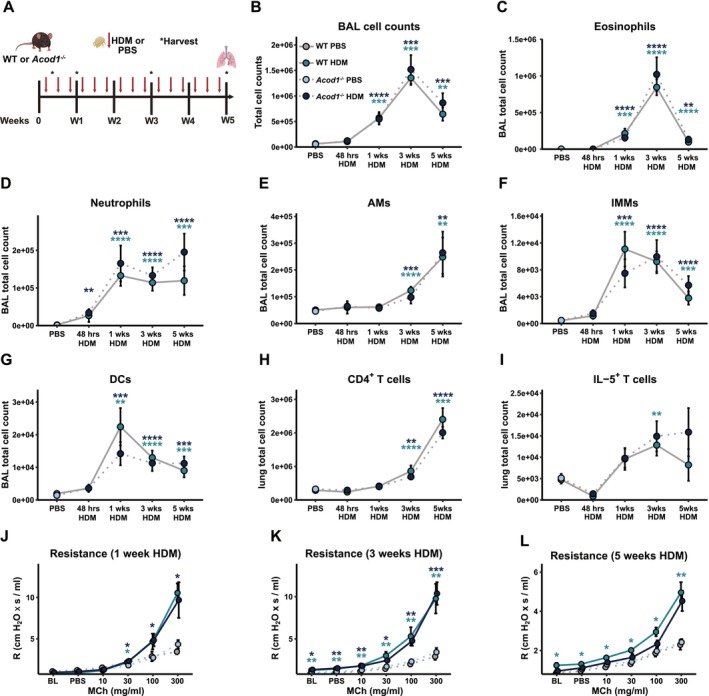
(A) *Acod1* ^*−/−*^ and WT control mice were exposed to inhaled HDM or PBS and BAL immune cell populations were assessed by flow cytometry 48 h after the first dose of HDM or one, three or five weeks of repeated HDM challenge. (B) Total BAL cell counts and absolute numbers of (C) eosinophils, (D) neutrophils, (E) AMs, (F) IMMs, (G) DCs, (H) lung CD4^+^, (I) CD4^+^IL‐5^+^. Airway resistance at one (J), three (K) or five (L) weeks of allergen challenge. Data shown from 1 to 2 independent experiments per timepoint with *n* = 3–8 mice per group per experiment. Significant differences between matched HDM‐ and PBS‐treated groups are indicated by stars in teal (WT PBS vs. WT HDM) or navy (*Acod1* ^*−/−*^ PBS vs. *Acod1* ^*−/−*^ HDM). Baseline PBS data shown as pooled from all timepoints. Data presented as mean ± SEM. Mann–Whitney test, *p < 0.05, **p < 0.01, ***p < 0.0001, ****p < 0.00001.

To determine the functional role of itaconate during AAD, we exposed WT or *Acod1* ^*−/−*^ mice which lack *Acod1* to inhaled HDM and assessed pulmonary cellular changes at 48 h, 1, 3, or 5 weeks post initial challenge (Figure 3A). Gating strategies for the identification of immune cell types in BAL and lung are detailed in Figure S3 and have been previously detailed [30]. In brief, live, CD45^+^ cells were selected and CD19^−^, CD90.2^−^ or NKp46^+^ cells deselected to identify CD64^+^SiglecF^+^CD11c^+^ AMs, SiglecF^+^CD64^−^CD11c^−^ eosinophils, and SiglecF^−^Ly6G^+^ neutrophils. SiglecF^−^Ly6G^−^ cells were further identified as MHCII^+^CD11c^+^CD103^+^ cDC1s, CD64^+^CD11b^+^CD103^−^ moDCs, or CD64^−^CD11b^+^CD103^−^ cDC2s, and the remaining MHC‐II^+/‐^CD11c^+/‐^CD11b^+^CD64^+^ cells assigned as IMMs. Using a separate staining panel, CD4 T cells were identified as live, CD45^+^CD3^+^CD8^−^CD4^+^ cells, and CD8 T cells were identified as live, CD45^+^CD3^+^CD4^−^CD8+ cells. CD4 T cell subsets were identified using intracellular staining for IL‐10, IL‐5, IL‐13, IL‐17, or IFN‐γ (Figure S3). Assessment of the cellular composition revealed no changes in total cell counts in BAL (Figure 3B) when comparing WT or *Acod1* ^*−/−*^ after 48 h, 1, 3, or 5 weeks of HDM exposure. Similarly, we detected no changes in proportions of eosinophils (Figure 3C), neutrophils (Figure 3D), AMs (Figure 3E), interstitial macrophages/monocytes (IMMs; Figure 3F), or dendritic cells (DCs; Figure 3G) when comparing WT to *Acod1* ^−/−^ mice. Similar patterns were observed in lung cell populations (Figure S4A–F). Additionally, we observed no impact of *Acod1* deficiency on numbers of CD4^+^ cells (Figure 3H), nor IL‐5^+^ (Figure 3I) T cells. *Acod1* deficiency did not influence lung function, as we observed no alteration in resistance (Figure 3J–L), elastance, or compliance parameters (Figure S4G–L) after 1, 3, or 5 weeks of allergen exposure in *Acod1* ^−/−^ mice compared to WT controls. Measurement of serum IgE (Figure S4M) and IgG1 (Figure S4N) indicated that there was no impact of *Acod1* deficiency on allergic sensitisation at any of the time points tested. In addition, we assessed the effect of *Acod1* deficiency on soluble mediators by multiplex assay and expression of disease‐relevant genes and found no differences between HDM‐treated WT or *Acod1* ^−/−^ mice (Figure S5A–J). Analysis of H&E and PAS stained lung sections indicated that airway lumen cell infiltration (Figure S5K) and mucus secretion (Figure S5L) were unaffected by *Acod1* deficiency. Finally, extracellular flux analysis indicated that AMs stimulated with HDM ex vivo were unaffected in *Acod1* ^−/−^ mice compared to controls (Figure  S5M–O). Together, these data indicate that *Acod1* deficiency does not affect key disease parameters of AAD, such as BAL and lung immune cell populations, serum antibody production, or AHR in a model of continuous HDM exposure.

### 3.4 Administration of Exogenous Itaconate to WT Mice Alters Innate Immune Cell Composition of the Airways

**FIGURE 4:**
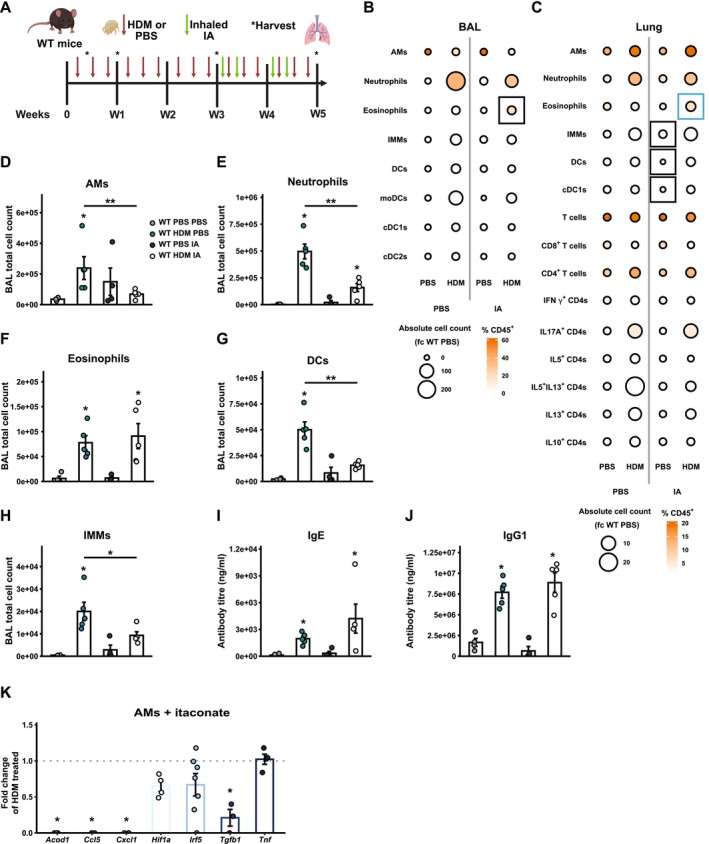
Exogenous itaconate alters innate immune cell composition (A) WT mice were exposed to inhaled HDM or PBS for five weeks and dosed with inhaled itaconate (IA) or PBS during the last two weeks of allergen challenge. Overview of all analysed immune cell populations in the (B) BAL and (C) lung. The size of the circle illustrates the absolute cell count as fold change relative to WT control mice receiving PBS for both treatments and the colour indicates the proportion of the respective cell type as percentage of all CD45^+^ cells. Significant changes in the proportion of CD45^+^ cells are highlighted by a black tile outline for comparisons between mice receiving PBS or IA as secondary treatment and blue tile outlines for comparison between mice receiving PBS or HDM. Numbers of (D) AMs, (E) neutrophils, (F) eosinophils, (G) DCs, (H) IMMs in BAL of HDM‐ or PBS‐exposed mice with or without IA treatment. Serum (I) IgE and (J) IgG1 in HDM‐exposed mice or controls. Data shown from one experiment with *n* = 4–5 mice per group. Significant differences between matched HDM‐ and PBS‐treated groups are indicated by stars above the HDM bar. (K) Gene expression of *Acod1*, *Ccl5*, *Hif1a*, *Irf5*, *Tgfb1* and *Tnf* in AMs ex vivo pre‐treted with itaconate followd by HDM exposure as fold change of control AMs treated with HDM. Data shown from two independent experiments with *n* = 3–4 mice per group. Significant changes compared to HDM‐treated controls are indicated by start above the bars. Data presented as mean ± SEM. Mann–Whitney test, *p < 0.05, **p < 0.01.

To investigate the effect of exogenous itaconate on the progression of AAD, WT mice were exposed to HDM for 5 weeks, with inhaled itaconate administered during the final 2 weeks of HDM exposure, and immune cell populations were analysed in BAL and lung tissue (Figure 4A–C). In HDM‐exposed mice, inhaled itaconate led to a significant reduction in absolute numbers of AMs (Figure 4D), neutrophils (Figure 4E), no change in eosinophils (Figure 4F), and reductions in total dendritic cells (DCs; Figure 4G) as well as interstitial macrophages (IMMs; Figure 4H). Despite these notable shifts in immune cell populations, no changes were observed in blood IgE (Figure 4I) or IgG1 (Figure 4J). Interestingly, in mice exposed to HDM for three weeks, itaconate did not alter any of the immune cell populations evaluated (Figure S6). We assessed the expression of key inflammatory chemokines, cytokines, and transcription factors involved in the regulation of macrophage phenotypes in primary murine AMs ex vivo treated with itaconate prior to HDM exposure (Figure 4K). Our data show that itaconate treatment significantly reduced the gene expression of *Acod1*, *Ccl5*, *Cxcl1*, and *Tgfb1*, but not *Hif1a*, interferon regulatory factor‐5 (*Irf5*), or *Tnf* (Figure 4K), consistent with its anti‐inflammatory effects.

## 4 Discussion

Accumulating evidence suggests that itaconate plays a potentially crucial role in orchestrating pulmonary immune responses and has emerged as a promising therapeutic target for the treatment of respiratory conditions. Although a role for itaconate has been explored in models of asthma in mice, the function of itaconate across specific human asthma phenotypes, as well as during human allergen exposure, has not yet been explored. Our study is, to our knowledge, the first to report alterations in levels of airway itaconate in human asthma.

Our work shows that allergen exposure in mild asthmatics led to a reduction of airway itaconate. In contrast, we observed no significant differences in baseline sputum itaconate levels among healthy controls and individuals with mild, moderate, or severe asthma. Notably, a recent study suggests that glucocorticoids can modulate macrophage phenotype by influencing the TCA cycle [32]. It is therefore possible that the use of corticosteroids by some patients potentially masked underlying differences in itaconate levels. Further investigation is warranted to assess the impact of corticosteroid treatment on the itaconate pathway. Of note, in the allergen challenge cohort at −24 h, one particularly high value for itaconate at 7 h was observed. This data point was retained in the analysis, as no clinical or biological rationale for its exclusion was identified. While its removal affected the statistical significance of certain metabolites (lactate and malate at 24 h, and itaconate at 7 h), the overall conclusions of the study remain unchanged.

It has recently been reported that *Acod1* deficiency promotes Th2 immune responses and airway inflammation induced by HDM in mice [23]. Of note, this study used an AAD with intraperitoneal administration of adjuvant with HDM, followed by three intranasal HDM challenges. Similarly, utilising a model with intranasal HDM sensitisation followed by HDM challenge, Li et al. [21] reported increased type 2 immunity and increased serum IgE in *Acod1* ^*−/−*^ mice compared to WT controls. In our model, disease severity was not impacted by *Acod1* deficiency, with AHR and innate immune cell populations comparable between WT and *Acod1* ^*−/−*^ mice. Notably, there are several key differences between our model and the one used in these studies, including the duration of allergen exposure, the supplier of HDM extract, and the HDM dosing strategy. The protocol employed by Li et al. for instance, may result in a greater induction of *Acod1* and itaconate than observed in our model. This suggests the presence of a threshold level of itaconate activity, above which itaconate begins to play a significant role. Supporting this idea, we found that in our model, exogenous itaconate mitigated several aspects of AAD, including neutrophilia. Although ACOD1 and itaconate are closely linked, ACOD1 deficiency may alter immune responses through both itaconate‐dependent and ‐independent mechanisms, which may explain the context‐dependent protective phenotypes observed in some infectious disease models. For example, ACOD1 plays contrasting roles in infectious disease models, providing antiviral protection in the context of Zika [33] and hepatitis B infection [34], but can also promote viral pathogenesis, as seen with respiratory syncytial virus [35] and vesicular stomatitis virus [36].

Recently, we reported that inhaled itaconate was protective in a murine model of lung fibrosis [19]. Our current study highlights the potential for itaconate, or itaconate analogues, as potential therapeutic tools for the treatment of asthma. Two recent studies reported that the administration of 4‐OI, a polar itaconate analogue, limited AAD in HDM as well as ovalbumin (OVA)‐induced models of AAD [23, 37]. While 4‐OI limited mitochondrial respiration in macrophages and mitochondrial stress in DCs, it furthermore reduced IL‐5 and IL‐13 levels via inhibition of the phosphorylation of STAT1 in CD4^+^ T cells. However, it has been reported that itaconate derivatives such as 4‐OI or dimethyl itaconate are not converted into intracellular itaconate and have distinct properties compared to endogenous itaconate [38, 39]. In our study, we exposed mice to ‘free’ itaconate via the inhaled route. Itaconate was delivered intranasally in a PBS vehicle; the rationale for using this delivery method was that this approach directly targets the airways. The selected dose was chosen based on our prior study, where we titrated dosing and timing, arriving at a dose that effectively modulates airway inflammation without inducing systemic/local effects [19]. In our experience, alternate‐day dosing is required to maintain efficacy, suggesting relatively rapid clearance or catabolism. Of note, although both *Acod1* expression and itaconate levels were elevated during HDM exposure, their dynamics were distinct. Notably, although the most pronounced change in *Acod1* expression was observed in AMs, *Acod1* is also expressed outside the AM compartment in the airways. Therefore, as *Acod1* is expressed in various lung cell types, itaconate production may be localised to certain regions of the lung or to specific cell populations, which could result in lower levels of itaconate being detected in the BAL fluid. Moreover, the dynamics of itaconate release from lung tissues into the BAL fluid may be influenced by factors such as cellular uptake, secretion, or degradation processes that are not fully captured by our current analysis. Itaconate catabolism remains incompletely understood; however, recent in vivo tracing studies show that free itaconate is rapidly catabolised into metabolites such as mesaconate, citramalate, and acetyl‐CoA, supporting its fast turnover and the need for frequent dosing to sustain bioactivity [40]. In our model, exogenous itaconate limited neutrophilia and Th2 cell numbers after five weeks of HDM exposure. However, we did not observe significant effects in itaconate‐treated mice at the three‐week time point. In the five‐week model, mice received four doses of itaconate during weeks four and five, while in the three‐week model, nine doses were given from week one. The differing outcomes may reflect the timing of treatment relative to disease progression, with itaconate having a greater impact when administered later, after endogenous levels have begun to accumulate. Consistent with our finding that exogenous itaconate limits type 2 responses, Li et al. [21] found that exogenous itaconate suppressed Th2 differentiation in vitro by reducing Gata3 expression and IL‐5/IL‐13 secretion in naïve CD4^+^ T cells. Thus, accumulating clinical and preclinical data suggest that insufficient or dysregulated itaconate production may contribute to allergic airway disease, and that restoring this pathway could represent a promising therapeutic strategy. These findings position itaconate not only as a biomarker of inflammation but as a potential metabolic checkpoint for intervention in Th2‐driven asthma.

In conclusion, our findings highlight a complex and context‐dependent role for itaconate in asthma. While allergen exposure alters itaconate levels, the absence of *Acod1* did not impact disease outcomes in mice. This discrepancy may stem from differences in experimental models, duration of allergen exposure, or compensatory mechanisms in the absence of *Acod1*. The therapeutic efficacy of inhaled itaconate suggests that exogenous modulation of this pathway could have clinical relevance, particularly in neutrophilic asthma phenotypes.

## Author Contributions

G.J.A. and A.J.B. designed the study; G.J.A., P.P.O., C.M., H.S., S.A.W., A.C., and J.M.H. carried out the work. All authors were involved in the interpretation of the results and in drafting and/or revising the manuscript, providing final approval, and vouching for the content of the final manuscript.

## Conflicts of Interest

A.J.B. has, via his institution, received consultancy fees or industry‐academic funding from Ammax Bio, Devpro Bio, GRI Bio, and Ionis Pharma. A.J.B. holds two patents relating to the use of itaconate or itaconate analogues for the treatment of fibrotic lung disease (PCT/GB2023/050629 and PCT/GB2020/052218). G.M.G. has received research funding from AstraZeneca, Biohaven, Genentech, Jasper Therapeutics, and Third Harmonics Bio paid to the institution. P.P.O. holds the patent related to the use of itaconate for the treatment of fibrotic lung disease (PCT/GB2020/052218).

## Supporting information


**Figure S1:** (A) Patients with mild asthma were challenged with sensitising allergen and induced sputum was collected at indicated timepoints. (B) Schematic of glycolysis and TCA cycle. (C) Heatmap showing sputum metabolites at 7 h and 24 h post allergen challenge. Levels are shown as log_2_ fold change compared to sputum metabolite level 24 h prior to allergen challenge. Levels of (D) Glucose, (E) Glycerol‐1‐phosphate, (F) Glycerol‐2‐phosphate, (G) pyruvate, (H) citrate, (I) cis‐aconitate, and (J) α‐ketogluterate in sputum supernatants pre and post allergen challenge, as determined by targeted GC–MS. *n* = 10 matched samples. Data presented as mean. (K) Sputum samples were obtained from healthy controls, or mild, moderate or severe asthmatics. Baseline levels of (L) citrate, (M) cis‐aconitate, (N) isocitrate, (O) itaconate in sputum supernatants, as determined by targeted GC–MS. *n* = 6–13 per group. Data presented as mean ± SEM. Mann–Whitney test, *p < 0.05, **p < 0.01.

**Figure S2:** Metabolite levels in BAL of WT or *Acod1* ^*−/−*^ mice exposed to inhaled HDM for (A) three or (B) five weeks as measured by targeted GC–MS.

**Figure S3:** Gating strategy for granulocytes, macrophages and Th2 cell populations.

**Figure S4:** all70107‐sup‐0004‐FigureS4.pdf. *Acod1* ^*−/−*^ and WT control mice were exposed to inhaled HDM or PBS and BAL immune cell populations were assessed by flow cytometry 48 h after the first dose of HDM or 1, 3 or 5 weeks of repeated HDM challenge.

**Figure S5:** Soluble mediators in the BAL of WT and *Acod1* ^*−/−*^ mice were measured after exposure to inhaled house dust mite (HDM) allergen for 3 weeks.

**Figure S6:** (A) WT mice were exposed to inhaled HDM or PBS and inhaled itaconate (IA) or PBS for 3 weeks.

**Table S1:** Patient inclusion and exclusion criteria (allergen challenge cohort).

## Data Availability

The data that support the findings of this study are available on request from the corresponding author. The data are not publicly available due to privacy or ethical restrictions.
