# Supplementary material for: Protective Role for Itaconate During Inhaled Allergen Challenge
Source: Allergy. 2025 Oct 24;81(4):1099–110. doi: 10.1111/all.70107 (PMC13040632; doi:10.1111/all.70107)
Supplement: Supplementary file 1 — Figure S1: (A) Patients with mild asthma were challenged with sensitising allergen and induced sputum was collected at indicated timepoints. (B) Schematic of glycolysis and TCA cycle. (C) Heatmap showing sputum metabolites at 7 h and 24 h post allergen challenge. Levels are shown as log2 fold change compared to sputum metabolite level 24 h prior to allergen challenge. Levels of (D) Glucose, (E) Glycerol‐1‐phosphate, (F) Glycerol‐2‐phosphate, (G) pyruvate, (H) citrate, (I) cis‐aconitate, and (J) α‐ketogluterate in sputum supernatants pre and post allergen challenge, as determined by targeted GC–MS. n = 10 matched samples. Data presented as mean. (K) Sputum samples were obtained from healthy controls, or mild, moderate or severe asthmatics. Baseline levels of (L) citrate, (M) cis‐aconitate, (N) isocitrate, (O) itaconate in sputum supernatants, as determined by targeted GC–MS. n = 6–13 per group. Data presented as mean ± SEM. Mann–Whitney test, *p < 0.05, **p < 0.01. [file ALL-81-1099-s001.pdf]

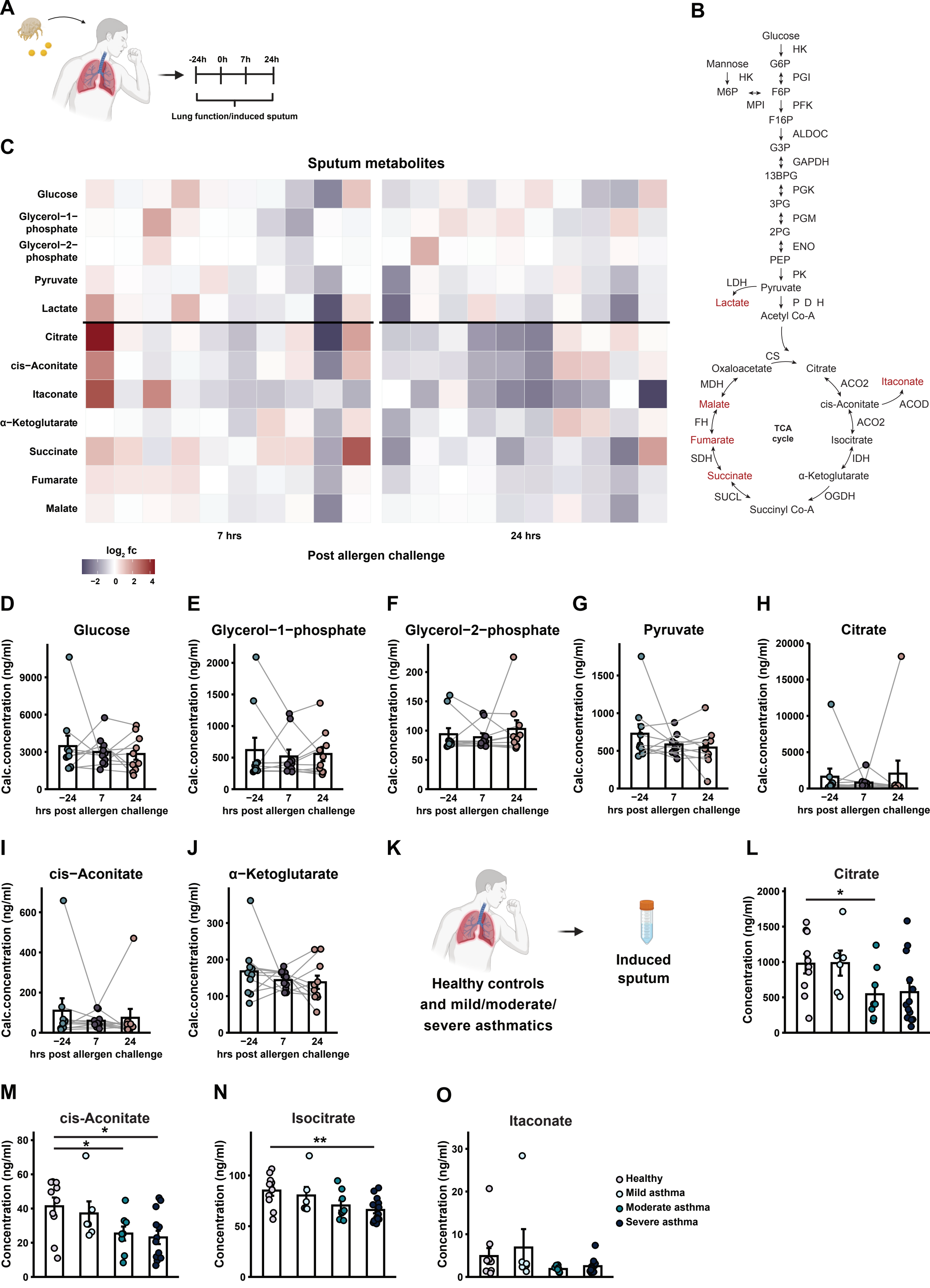

Figure S1. (A) Patients with mild asthma were challenged with sensitising allergen and induced sputum was collected at indicated timepoints. (B) Schematic of glycolysis and TCA cycle. (C) Heatmap showing sputum metabolites at 7 hrs and 24 hrs post allergen challenge. Levels are shown as $\log_2$ fold change compared to sputum metabolite level 24 hrs prior to allergen challenge. Levels of (D) Glucose, (E) Glycerol-1-phosphate, (F) Glycerol-2-phosphate, (G) pyruvate, (H) citrate, (I) cis-aconitate, and (J) α-ketogluterate in sputum supernatants pre and post allergen challenge, as determined by targeted GC-MS. n = 10 matched samples. Data presented as mean. (K) Sputum samples were obtained from healthy controls, or mild, moderate or severe asthmatics. Baseline levels of (L) citrate, (M) cis-aconitate, (N) isocitrate, (O) itaconate in sputum supernatants, as determined by targeted GC-MS. n = 6 - 13 per group. Data presented as mean ± S.E.M. Mann-Whitney test, * $p < 0.05$, ** $p < 0.01$.
