# Supplementary material for: Protective Role for Itaconate During Inhaled Allergen Challenge
Source: Allergy. 2025 Oct 24;81(4):1099–110. doi: 10.1111/all.70107 (PMC13040632; doi:10.1111/all.70107)
Supplement: Supplementary file 2 — Figure S2: Metabolite levels in BAL of WT or Acod1 −/− mice exposed to inhaled HDM for (A) three or (B) five weeks as measured by targeted GC–MS. [file ALL-81-1099-s003.pdf]

A

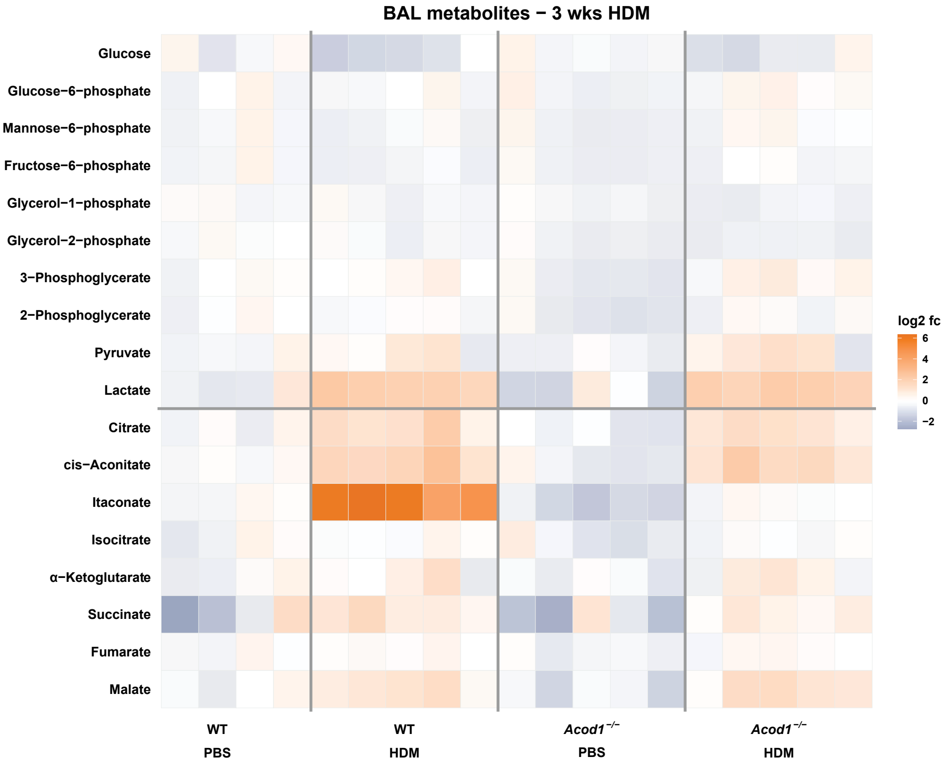

B

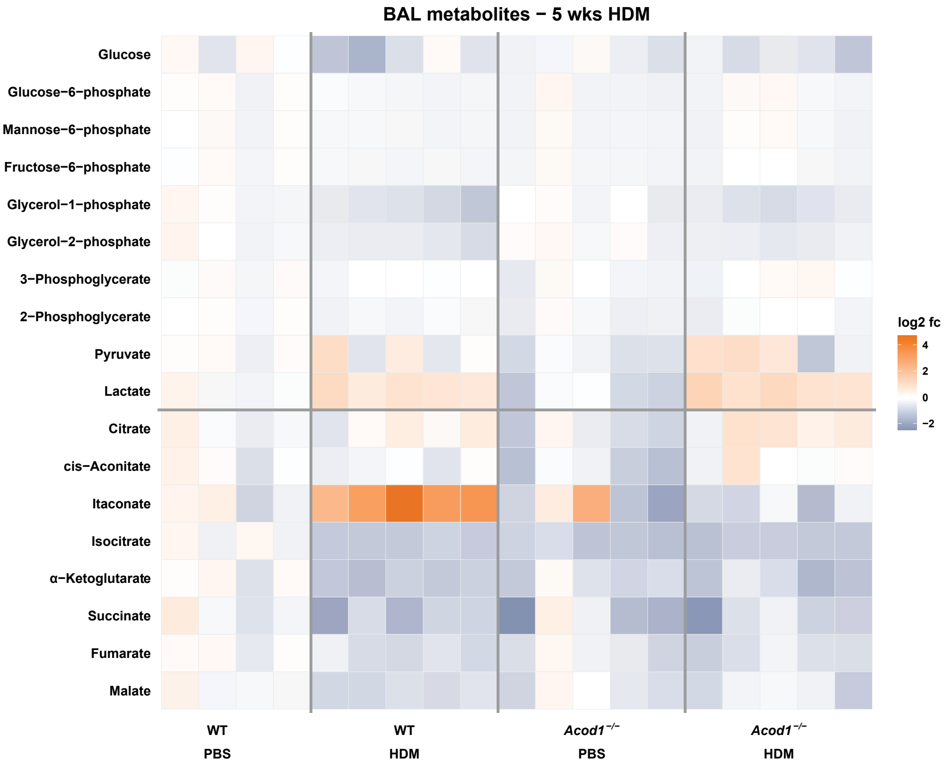

Figure S2. (A) Schematic of HDM exposure model. Metabolite levels in BAL of WT or *Acod1*$^{-/-}$ mice exposed to inhaled HDM for (A) three or (B) five weeks as measured by targeted GC-MS. Levels are shown as $\log_2$ fold change compared to WT PBS control mice. Data shown from one experiment per timepoint with n = 4 - 5 mice per group per experiment.
