# Supplementary material for: Protective Role for Itaconate During Inhaled Allergen Challenge
Source: Allergy. 2025 Oct 24;81(4):1099–110. doi: 10.1111/all.70107 (PMC13040632; doi:10.1111/all.70107)
Supplement: Supplementary file 3 — Figure S3: Gating strategy for granulocytes, macrophages and Th2 cell populations. [file ALL-81-1099-s006.pdf]

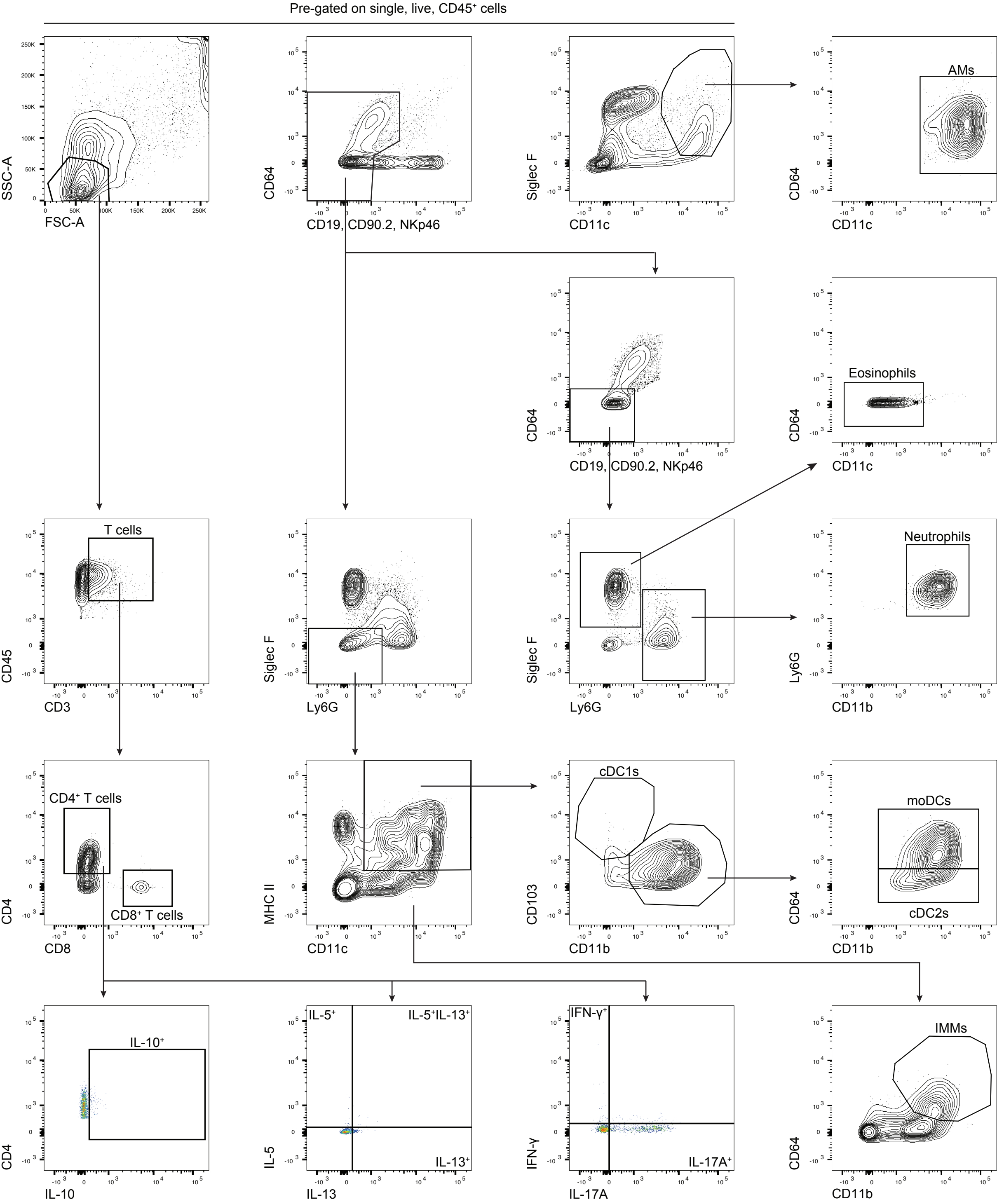

Figure S3. Gating strategy for granulocytes, macrophages and Th2 cell populations. Representative flow cytometry plots of lung cell suspensions from a WT mouse exposed to inhaled HDM for three weeks.
