# Supplementary material for: Protective Role for Itaconate During Inhaled Allergen Challenge
Source: Allergy. 2025 Oct 24;81(4):1099–110. doi: 10.1111/all.70107 (PMC13040632; doi:10.1111/all.70107)
Supplement: Supplementary file 4 — Figure S4: all70107‐sup‐0004‐FigureS4.pdf. Acod1 −/− and WT control mice were exposed to inhaled HDM or PBS and BAL immune cell populations were assessed by flow cytometry 48 h after the first dose of HDM or 1, 3 or 5 weeks of repeated HDM challenge. [file ALL-81-1099-s007.pdf]

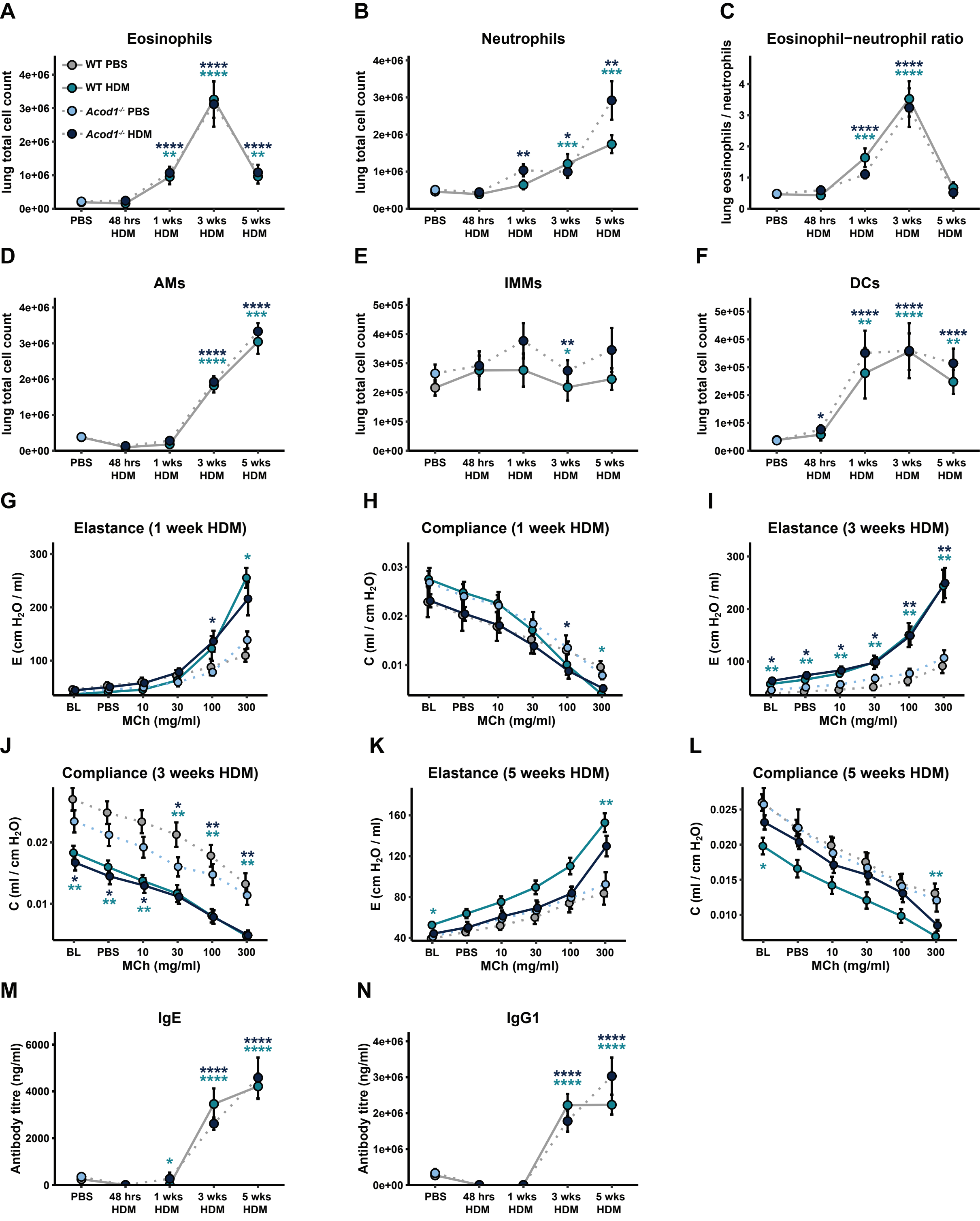

Figure S4. *Acod1*$^{-/-}$ and WT control mice were exposed to inhaled HDM or PBS and BAL immune cell populations were assessed by flow cytometry 48 hrs after the first dose of HDM or one, three or five weeks of repeated HDM challenge. Absolute numbers of eosinophils (A), neutrophils (B) in lung tissue, eosinophil:neutrophil ratio (C) in lung tissue. Absolute numbers (D) AMs, (E) IMMs and (F) DCs, in lung tissue. Airway elastance after (G) one, (I) three or (K) five weeks of allergen challenge. Airway compliance after (H) one, (J) three or (L) five weeks of allergen challenge. Total serum (M) IgE (M) and (N) IgG1 (N) after 48 hrs, one, three or five weeks of allergen challenge. Significant differences between matched HDM- and PBS-treated groups are indicated by stars in teal (WT PBS vs WT HDM) or navy (*Acod1*$^{-/-}$ PBS vs *Acod1*$^{-/-}$ HDM). Data shown from 1 - 2 independent experiments per timepoint with n = 3 - 8 mice per group per experiment. Baseline PBS data shown as pooled from all timepoints. Data presented as mean ± S.E.M. Mann-Whitney test, * $p < 0.05$, ** $p < 0.01$, *** $p < 0.0001$, **** $p < 0.00001$.
