# Supplementary material for: Protective Role for Itaconate During Inhaled Allergen Challenge
Source: Allergy. 2025 Oct 24;81(4):1099–110. doi: 10.1111/all.70107 (PMC13040632; doi:10.1111/all.70107)
Supplement: Supplementary file 5 — Figure S5: Soluble mediators in the BAL of WT and Acod1 −/− mice were measured after exposure to inhaled house dust mite (HDM) allergen for 3 weeks. [file ALL-81-1099-s005.pdf]

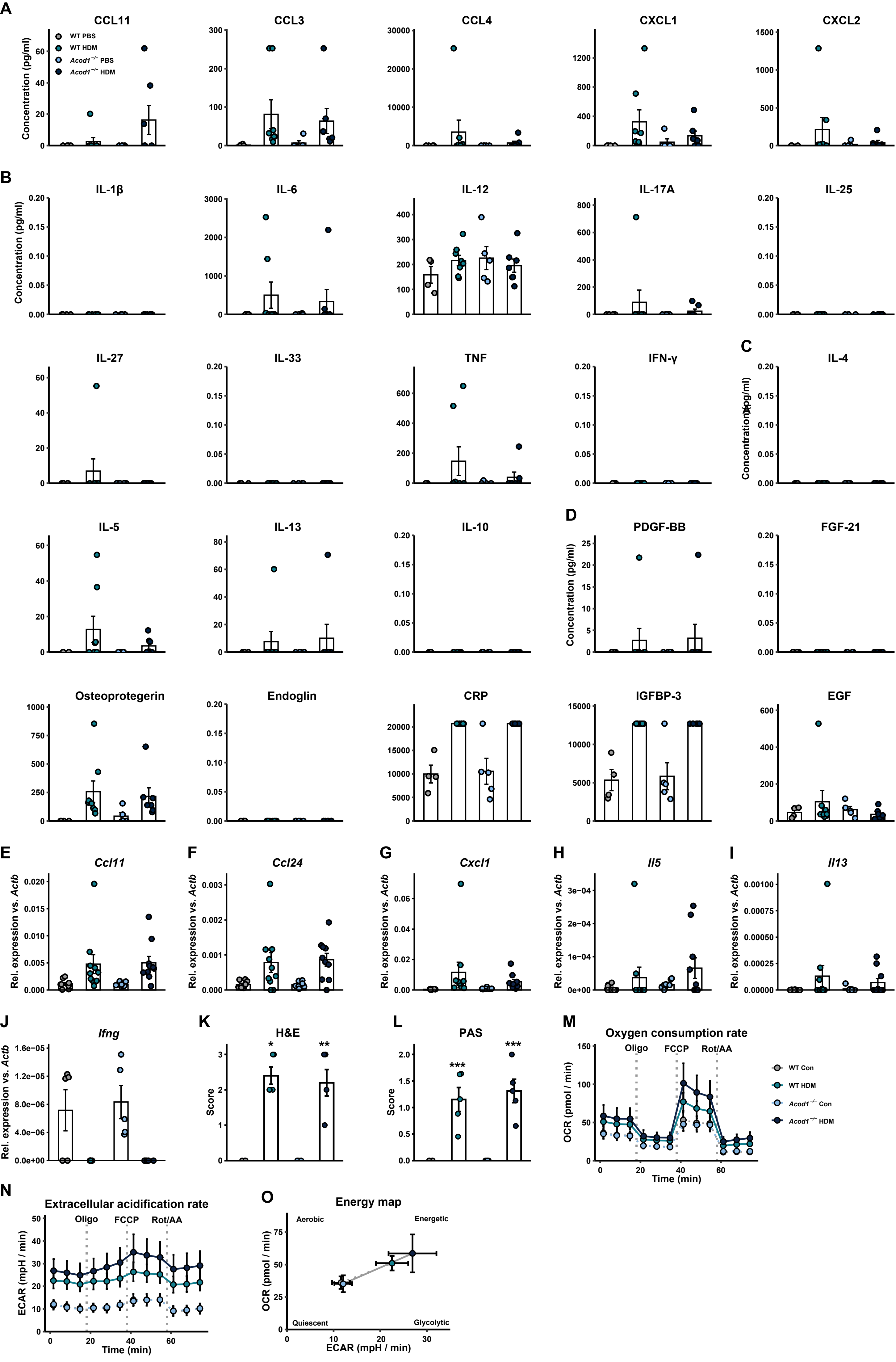

Figure S5. Soluble mediators in the BAL of WT and *Acod1*$^{-/-}$ mice were measured after exposure to inhaled house dust mite (HDM) allergen for three weeks. Protein levels were quantified using a Luminex multiplex assay. Results are shown as pg/ml BAL for the following groups: (A) chemokines (CCL11, CCL3, CCL4, CXCL1, CXCL2), (B) pro-inflammatory cytokines (IL-1β, IL-6, IL-12, IL-17A, IL-25, IL-27, IL-33, TNF, IFN-γ) or Th2 cytokines (IL-4, IL-5, IL-13) and the immuno-regulatory cytokine (IL-10), (D) Growth factors and other markers (PDGF-BB, FGF-21, Osteoprotegerin, Endoglin, CRP, IGFBP-3, EGP). Data are pooled from two independent experiments with n = 2 - 5 mice per group per experiment. Gene expression of (E) *Ccl11*, (F) *Ccl24*, (G) *Cxcl1*, (H) *Il5*, (I) *Il13* or (J) *Ifng* in whole lung homogenates WT and *Acod1*$^{-/-}$ HDM-exposed mice. Paraffin-embedded lung sections of WT and *Acod1*$^{-/-}$ mice with HDM-induced AAD or control mice were stained with (K) H&E to visualise immune cell infiltration or (L) Periodic-Acid Schiff as a marker for mucus production. Data shown from one experiment with n = 4 - 5 mice per group per experiment. AMs obtained from BAL of naïve WT and *Acod1*$^{-/-}$ mice were *ex vivo* exposed to HDM for 24 hrs. (M) Oxygen consumption rate (OCR) and (N) extracellular acidification rate (ECAR) were measured during Cell Mito Stress Test in response to the mitochondrial adenosine triphosphate (ATP)-synthase inhibitor oligomycin (Oligo), the mitochondrial uncoupler carbonyl cyanide-4-(trifluoromethoxy)phenylhydrazone (FCCP) and inhibitors of the electron transport chain complexes I and III, rotenone and anti-mycin A (Rot + AA). (O) Energy map showing the overall energetic phenotype of the cells indicating four different energetic states: quiescent, glycolytic, aerobic and energetic. Data pooled from two independent experiments with n = 4 - 5 mice per group. Data presented as mean ± S.E.M. Mann-Whitney test, * $p < 0.05$, ** $p < 0.01$, *** $p < 0.0001$.
