# Supplementary material for: Protective Role for Itaconate During Inhaled Allergen Challenge
Source: Allergy. 2025 Oct 24;81(4):1099–110. doi: 10.1111/all.70107 (PMC13040632; doi:10.1111/all.70107)
Supplement: Supplementary file 6 — Figure S6: (A) WT mice were exposed to inhaled HDM or PBS and inhaled itaconate (IA) or PBS for 3 weeks. [file ALL-81-1099-s004.pdf]

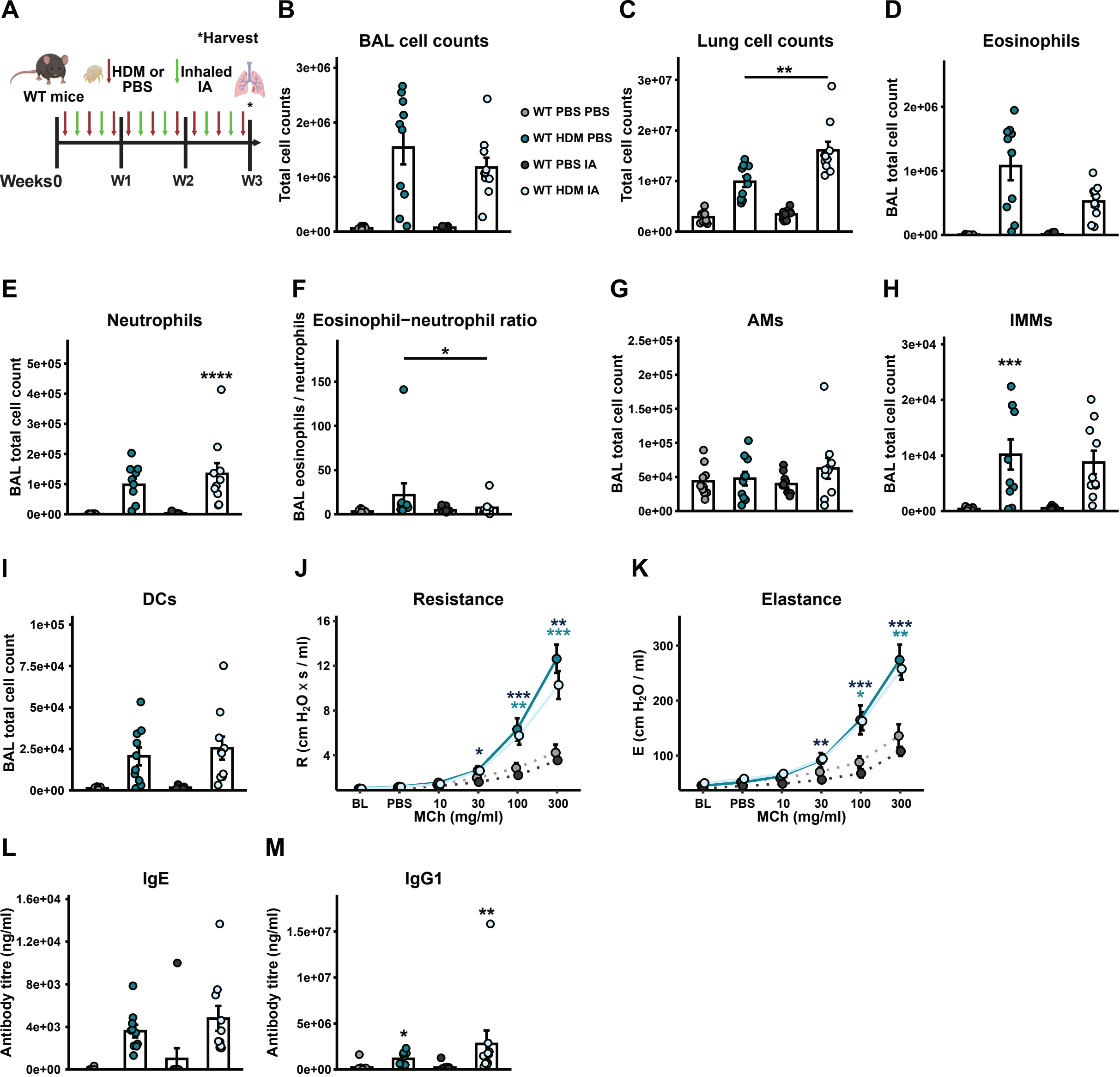

Figure S6. (A) WT mice were exposed to inhaled HDM or PBS and inhaled itaconate (IA) or PBS for three weeks. Cell counts in the (B) BAL and (C) lung. Numbers of (D) eosinophils, (E) neutrophils, (F) eosinophil:neutrophil ratio, (G) numbers of AMs, (J) IMMs (H), DCs (I) in BAL of HDM- or PBS-exposed mice with or without IA treatment. Significant differences between matched HDM- and PBS-treated groups are indicated by stars above the HDM bar. Airway resistance and (K) elastance. Significant differences between matched HDM- and PBS-treated groups are indicated by stars in teal (HDM PBS vs PBS PBS) or light blue (HDM IA vs PBS IA). Serum (L) IgE and (M) IgG1 in HDM exposed mice or controls. Significant differences between matched HDM- and PBS-treated groups are indicated by stars above the HDM bar. Data pooled from two independent experiments with n = 4 - 5 mice per group per experiment. Data presented as mean ± S.E.M. Mann-Whitney test, * $p < 0.05$, ** $p < 0.01$, *** $p < 0.0001$.
