# Supplementary material for: Protective Role for Itaconate During Inhaled Allergen Challenge
Source: Allergy. 2025 Oct 24;81(4):1099–110. doi: 10.1111/all.70107 (PMC13040632; doi:10.1111/all.70107)
Supplement: Supplementary file 7 — Table S1: Patient inclusion and exclusion criteria (allergen challenge cohort). [file ALL-81-1099-s002.docx]

**Supplementary Tables**

| **Inclusion criteria** | **Exclusion criteria** |
| --- | --- |
| General good health | Worsening asthma or recent (<6 weeks) respiratory tract infection |
| Mild to moderate asthma; stable, allergic asthma; history of episodic wheeze and shortness of breath | Pregnancy, lactating, or positive serum pregnancy test |
| Baseline FEV 1 % predicted ≥70% | Recent (<28 days) use of corticosteroids, immunosuppressive drugs, anticoagulants |
| Baseline PC20(methacholine) ≤16mg/ml | Recent use of nonsteroidal anti-inflammatory drugs (<24 hours) or aspirin (<7 days) |
| Positive skin-prick test to common aeroallergens | Chronic use of medication for treatment of allergic lung disease other than short-acting β2-agonists |
| Positive allergen-induced early airway bronchoconstriction (~~FEV 1 % predicted <70%~~ drop in FEV 1 of 20%) | Use of caffeine-containing products or medications (<12 hours), or alcohol or over-the-counter drugs (<48 hours), or inhaled bronchodilators (<8 hours) prior to the methacholine and allergen challenges |
| Able to understand and give written informed consent | Use of tobacco products of any kind (<1 year), or smoking history of >10 packs per year |
|  | Presence of lung disease other than mild to moderate allergic asthma |
|  | Recent (<1 year) history of alcohol dependency |
|  | Unwillingness or inability to comply with the study protocol for any other reason |

**Table S1. Patient inclusion and exclusion criteria (allergen challenge cohort).**
